# The Effect of TBB, as an Initiator, on the Biological Compatibility of PMMA/MMA Bone Cement

**DOI:** 10.3390/ijms21114016

**Published:** 2020-06-04

**Authors:** Kosuke Hamajima, Ryotaro Ozawa, Juri Saruta, Makiko Saita, Hiroaki Kitajima, Samira Rahim Taleghani, Dan Usami, Donya Goharian, Mitsunori Uno, Ken Miyazawa, Shigemi Goto, Keiichi Tsukinoki, Takahiro Ogawa

**Affiliations:** 1 Weintraub Center for Reconstructive Biotechnology, Division of Advanced Prosthodontics, UCLA School of Dentistry, Los Angeles, CA 90095-1668, USA; hamajima.k0329@gmail.com (K.H.); ozrt1021@gmail.com (R.O.); saruta@kdu.ac.jp (J.S.); saita@kdu.ac.jp (M.S.); hiroaki_k_0315@yahoo.co.jp (H.K.); s.r.taleghani@gmail.com (S.R.T.); danusami1999@g.ucla.edu (D.U.); goharian.donya@gmail.com (D.G.); uno@dent.asahi-u.ac.jp (M.U.); 2 Department of Orthodontics, School of Dentistry, Aichi Gakuin University, 1-1-100 Kusumoto-cho, Chikusa-ku, Nagoya, Aichi 464-8650, Japan; miyaken@dpc.agu.ac.jp (K.M.); shig@dpc.aichi-gakuin.ac.jp (S.G.); 3 Department of Oral Interdisciplinary Medicine (Prosthodontics & Oral Implantology), Graduate School of Dentistry, Kanagawa Dental University, 82 Inaoka, Yokosuka, Kanagawa 238-8580, Japan; 4 Department of Oral Science, Graduate School of Dentistry, Kanagawa Dental University, 82 Inaoka, Yokosuka, Kanagawa 238-8580, Japan; tsukinoki@kdu.ac.jp; 5 Department of Oral and Maxillofacial Surgery, Graduate School of Medicine, Yokohama City University, 3-9 Fukuura, Kanazawa-ku, Yokohama, Kanagawa 236-0004, Japan; 6 Department of Prosthodontics, Division of Oral Functional Science and Rehabilitation, Asahi University School of Dentistry, 1851-1 Hozumi, Mizuho, Gifu 501-0296, Japan

**Keywords:** arthroplasty, balloon kyphoplasty, cytotoxicity, free radical, hydrophilic, implants, orthopedic surgery, total hip replacement

## Abstract

Acrylic bone cement is widely used in orthopedic surgery for treating various conditions of the bone and joints. Bone cement consists of methyl methacrylate (MMA), polymethyl methacrylate (PMMA), and benzoyl peroxide (BPO), functioning as a liquid monomer, solid phase, and polymerization initiator, respectively. However, cell and tissue toxicity caused by bone cement has been a concern. This study aimed to determine the effect of tri-n-butyl borane (TBB) as an initiator on the biocompatibility of bone cement. Rat spine bone marrow-derived osteoblasts were cultured on two commercially available PMMA-BPO bone cements and a PMMA-TBB experimental material. After a 24-h incubation, more cells survived on PMMA-TBB than on PMMA-BPO. Cytomorphometry showed that the area of cell spread was greater on PMMA-TBB than on PMMA-BPO. Analysis of alkaline phosphatase activity, gene expression, and matrix mineralization showed that the osteoblastic differentiation was substantially advanced on the PMMA-TBB. Electron spin resonance (ESR) spectroscopy revealed that polymerization radical production within the PMMA-TBB was 1/15–1/20 of that within the PMMA-BPO. Thus, the use of TBB as an initiator, improved the biocompatibility and physicochemical properties of the PMMA-based material.

## 1. Introduction

The prevalence of skeletal diseases and disorders is on a sharp increase, due to the ageing population. Polymethyl methacrylate (PMMA)-based bone cements are widely used for treating these conditions [1,2,3,4,5]. Specifically, they are used to fix metallic implants and restore fractured or diseased spines, bones, and joints. PMMA-based bone cements consist of PMMA powder, methyl methacrylate (MMA) liquid, and benzoyl peroxide (BPO), which acts as a polymerization initiator. Currently, commercially available bone cements have two clinical issues: implant failure and systemic complications [6,7,8,9,10]. Due to the inevitable toxic effects of bone cement materials thus far, cells and tissues around the cement often undergo pathogenic and necrotic changes, resulting in inflammation, bone resorption, and eventually implant loosening, when used with a metallic implant [7,11,12,13,14,15,16,17]. A revision surgery is required within 20 years after first surgery in 15% of total hip replacement cases, and even worse, when the surgery involves patients younger than 70 years, the lifetime risk of revision surgery rises to 35% [18]. The systemic complications are collectively defined as bone cement implantation syndrome (BCIS) [6,12,13,19]. Patients who undergo orthopedic surgery using bone cement suffer from perioperative or postoperative complications, such as hypoxia, hypotension, cardiac arrhythmias, increased pulmonary vascular resistance, cardiac arrest, or a combination of these [6,12,13,19]. The incidence of BCIS is as high as 28% depending on the part of the body where the surgery occurred [20] and can lead to unexpected death in a certain percentage of patients [12,21].

Many studies have been conducted to explain the mechanisms behind the toxic effects of bone cements and explore potential solutions. Oxidative stress induced by the production of free radicals during PMMA polymerization may be a primary cause [7,17,22,23]. Although radical or non-radical reactive oxygen species (ROS) are important components in various metabolic activities in the human body, they are harmful and trigger inflammatory reactions and functional damages at the cellular and tissue levels [24,25,26], once it is overproduced or when an imbalance between the ROS and antioxidant redox system is triggered. Specifically, free radicals and oxidative stress derived from bone cements lead to a significant reduction in the cell viability, proliferation, differentiation, and mineralization of osteoblasts [25,26,27,28,29,30]. To mitigate this problem, an effective measure was introduced to neutralize free radicals. Adding an antioxidant amino acid, N-acetyl-cysteine (NAC), to PMMA materials scavenged the radicals and significantly reduced the cytotoxicity in a dose-dependent manner [7,17,22,23].

The second problematic property of bone cements is their exothermic reaction during polymerization. Heat is generated from cleavage of a carbon double-bond to a single bond. The temperatures within polymerizing bone cements may reach 75 to 95 °C [31,32,33,34], which causes focal bone necrosis [35,36] and local interference in blood circulation. Addition of chitosan and chitosan/graphene oxide nanocomposite powders successfully lowered the polymerization temperature by more than 10 °C, resulting in the increased survival of osteoblastic cells cultured on the bone cement [37].

The third drawback of bone cements leading to its toxicity is the residual monomer after polymerization. The immediate or gradual release of unreacted monomers is cytotoxic and tissue toxic [38]. N,N-dimethyl-p-toluidine (DmpT) has been proven to decrease the amount of residual monomer by increasing the completeness of polymerization and is used in commercial bone cement products as a polymerization activator or co-initiator with BPO [39,40,41]. Optimizing the DmpT concentration is difficult and dependent on the various conditions of other ingredients [39,41,42]. The adverse chemical effects of the residual monomers in current bone cements remain to be addressed. In addition, there is a chemical dilemma that there is a greater increase in temperature with more complete polymerization [42,43].

Although the above-mentioned additive measures to counteract the negative physicochemical properties of bone cements were effective to improve its biocompatibility, cytotoxicity and clinical complications still remain significant concerns, due to the fundamental reaction and behavior of PMMA polymerization [8,21,32,44,45,46]. In addition, adding ingredients can be an additional source of toxicity, like in the case of residual DmpT [41,47]. In this study, we hypothesize that replacing a polymerization initiator without altering or adding to PMMA and MMA, can lead to a significant improvement in bone cement biocompatibility. Therefore, we tested tri-n-butyl borane (TBB) as a new initiator, since it has been used in the development of dental adhesive materials [48,49]. The objective of this study was to examine the biological compatibility of an experimental bone cement material made of PMMA/MMA-TBB, compared to two commercially available PMMA/MMA-BPO bone cements.

## 2. Results

### 2.1. Improved Initial Attachment of Osteoblasts on PMMA-TBB

**Figure 1:**
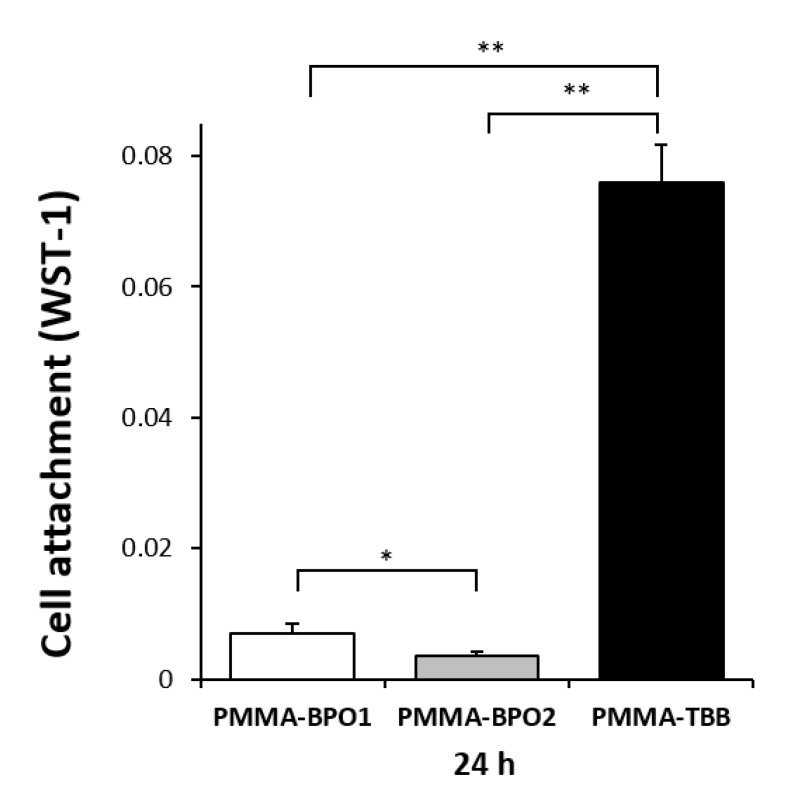
Attachment of osteoblasts on the three different bone cement surfaces during the initial stage of culture. The number of cells attached to each material surface during a 24-h incubation, evaluated using a water-soluble tetrazolium salts-1 (WST-1) assay. Each value represents the mean ± standard deviation of triplicate experiments (*n* = 3). * *p* < 0.05, ** *p* < 0.01, one-way ANOVA followed by a Bonferroni test.

The initial cell attachment was assessed by the number of osteoblasts attached to the three different bone cement surfaces after a 24-h culture using water-soluble tetrazolium salts-1 (WST-1) assay. The number of cells attached to the PMMA-BPO1 surface was significantly higher than that attached on the PMMA-BPO2 surface (Figure 1). More importantly, even more cells were attached to the PMMA-TBB surface than the PMMA-BPO1 and PMMA-BPO2 surfaces. The osteoblast attachment on PMMA-TBB was approximately 10 and 20 times greater than those of PMMA-BPO1 and PMMA-BPO2, respectively.

### 2.2. Improved Osteoblast Proliferation on PMMA-TBB

**Figure 2:**
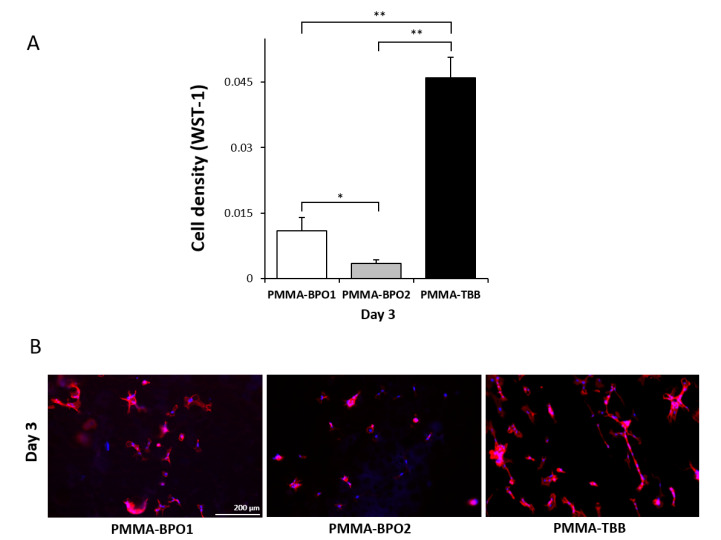
Proliferation of osteoblasts on the three different bone cement surfaces during the mid-stage of the culture. (**A**) The number of cells attached to the surface of each material at day 3 of incubation, evaluated using a WST-1 assay. (**B**) Visualized osteoblasts 3 days after seeding on the material. Representative fluorescence microscopy images of cells stained with rhodamine phalloidin for actin filaments (red) and DAPI for nucleus (blue). Each value represents the mean ± standard deviation of triplicate experiments (*n* = 3). * *p* < 0.05, ** *p* < 0.01, one-way ANOVA followed by a Bonferroni test. Scale bar = 200 µm.

To assess the proliferative activity of osteoblasts after settling, WST-1 assay was continued on day 3 after seeding. The number of propagated cells was higher in the order of the PMMA-TBB, PMMA-BPO1, and PMMA-BPO2, revealing a more biocompatible local environment on the PMMA-TBB continuing from day 1 to 3 (Figure 2A). The results of the WST-1 assay were confirmed using a fluorescent microscopic observation showing a greater number of cells on the PMMA-TBB than on the PMMA-BPO cements (Figure 2B). Cells appeared to be elongated, colonized, and networked on the PMMA-TBB, whereas they appeared scattered and isolated from each other on the PMMA-BPO materials.

### 2.3. Enhanced Spreading Behavior of Osteoblasts on PMMA-TBB

**Figure 3:**
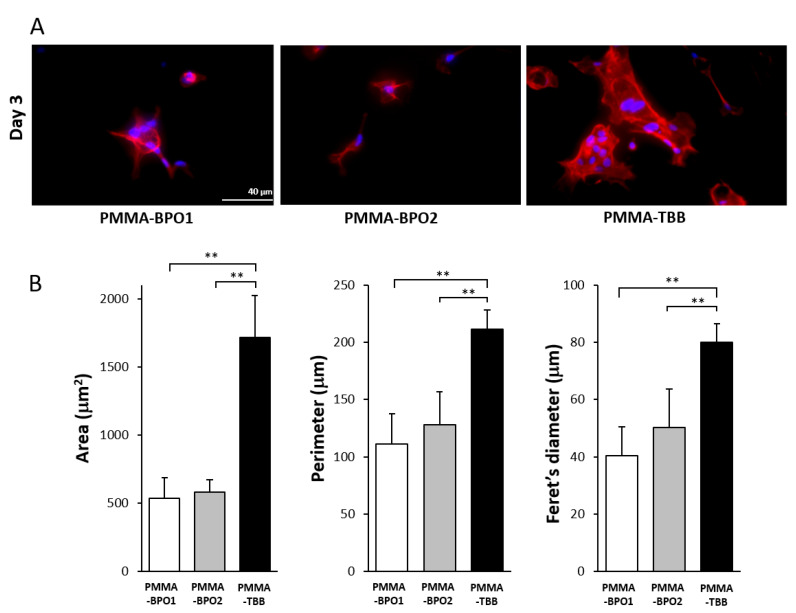
Representative high-magnification fluorescence microscopy images of the spreading osteoblasts 3 days after seeding on three different bone cement surfaces. (**A**) Fluorescence microscopic images of osteoblast following immunochemical staining for cytoskeletal actin (red) and nucleus (blue). Scale bar = 40 µm. (**B**) Histograms for cytomorphometric parameters measured from the images. Each value represents the mean ± standard deviation of six measurements (*n* = 6). ** *p* < 0.01, one-way ANOVA followed by a Bonferroni test. Scale bar = 40 µm.

We continued microscopic observation using high-magnification images on day 3. Osteoblasts on the PMMA-TBB were apparently larger than those on the two other bone cements, having a spindle shape, with more developed cyto-projections and intense expression of cyto-skeletal actin (Figure 3A). In addition, an overlapping morphology of cells with multiple nuclei was seen on the PMMA-TBB, indicating the advance of cellular colonization/proliferation. These qualitative observations on cellular behaviors were confirmed using cytomorphometry, showing significantly higher values for the area, perimeter, and Feret’s diameter of the cells on PMMA-TBB than on the PMMA-BPO materials (Figure 3B).

### 2.4. Improved Osteoblastic Functional Phenotype on PMMA-TBB

**Figure 4:**
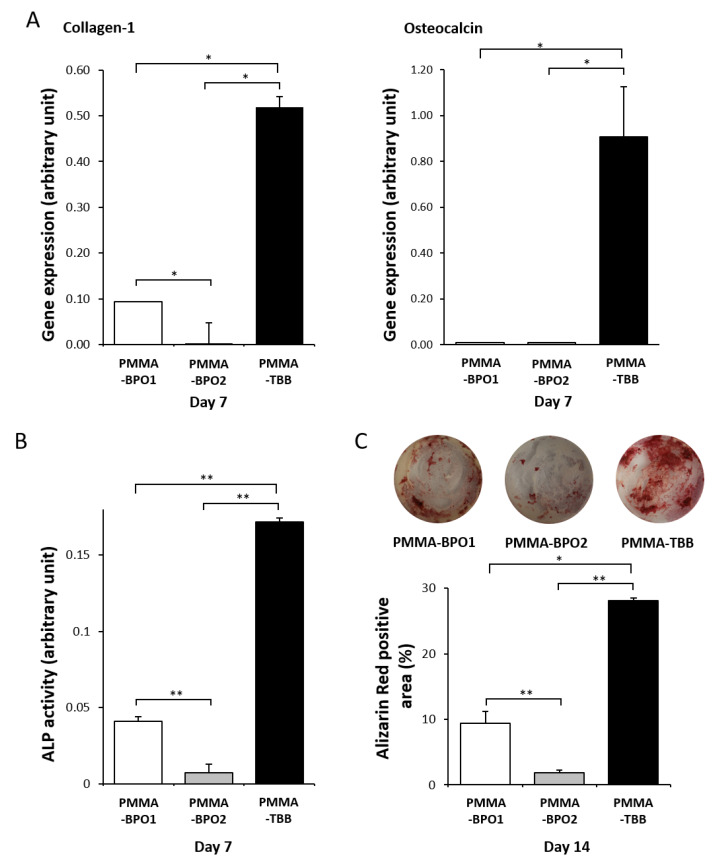
Biological characteristics of osteoblasts on three different bone cement surfaces. (**A**) Real-time qPCR analysis of mRNA expression of bone-related genes of collagen type I alpha 1 (collagen-1) and osteocalcin on the three different materials on day 7 using osteoblastic cell cultures. Relative expression levels (2^−ΔΔCt^ values) of the genes of interest were normalized to that of the housekeeping gene *Gapdh*. (**B**) Alkaline phosphatase (ALP) activity at day 7, colorimetrically quantified and standardized relative to cell number. (**C**) Representative images of mineral deposition evaluated using Alizarin red staining at culture day 14 (top). The histogram of the percentage of the Alizarin-positive area relative to total cell growth area on a culture well on the same day (bottom). Each value represents the mean ± standard deviation of triplicate experiments (*n* = 3). * *p* < 0.05, ** *p* < 0.01, one-way ANOVA followed by a Bonferroni test.

Furthermore, we examined how functional differentiation and phenotypes of osteoblasts are influenced on the three different materials. On day 7 of culture, quantitative PCR showed that the expression of collagen typeⅠalpha 1 (collagen-1) was significantly higher on the PMMA-TBB than on the PMMA-BPO1 and PMMA-BPO2 materials (Figure 4A). A similar result was obtained on the expression of the osteocalcin gene.

Alkaline phosphatase (ALP) activity, an early-to-mid stage maker, measured on day 7, was also significantly higher on the PMMA-TBB than on both the PMMA-BPO cements (Figure 4B). Lastly, the matrix mineralization, a late-stage maker of osteoblastic differentiation, evaluated using alizarin red stain on day 14, was significantly higher on the PMMA-TBB than on the two PMMA-BPO cements (Figure 4C).

### 2.5. Physicochemical Properties of the PMMA-TBB Material

**Figure 5:**
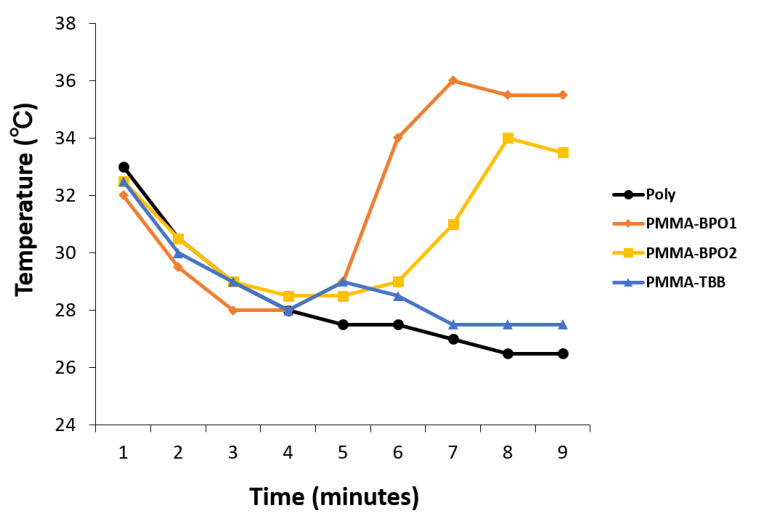
Temperature changes of the three different materials during polymerization. The measurement was continued until the temperature reached a peak in all materials.

The degree of heat generation during polymerization was assessed by measuring the temperature of the water where the bone cement material was immersed. The temperature peaked at 34 to 36 °C for PMMA-BPO bone cements approximately 7 to 8 min after mixing, whereas the temperature remained as low as 29 °C for the PMMA-TBB without a typical spike (Figure 5). After 9 min, the temperature remained at 33 °C or higher around the PMMA-BPO cements without a clear downturn, while the temperature dropped below 28 °C around the PMMA-TBB material.

**Figure 6:**
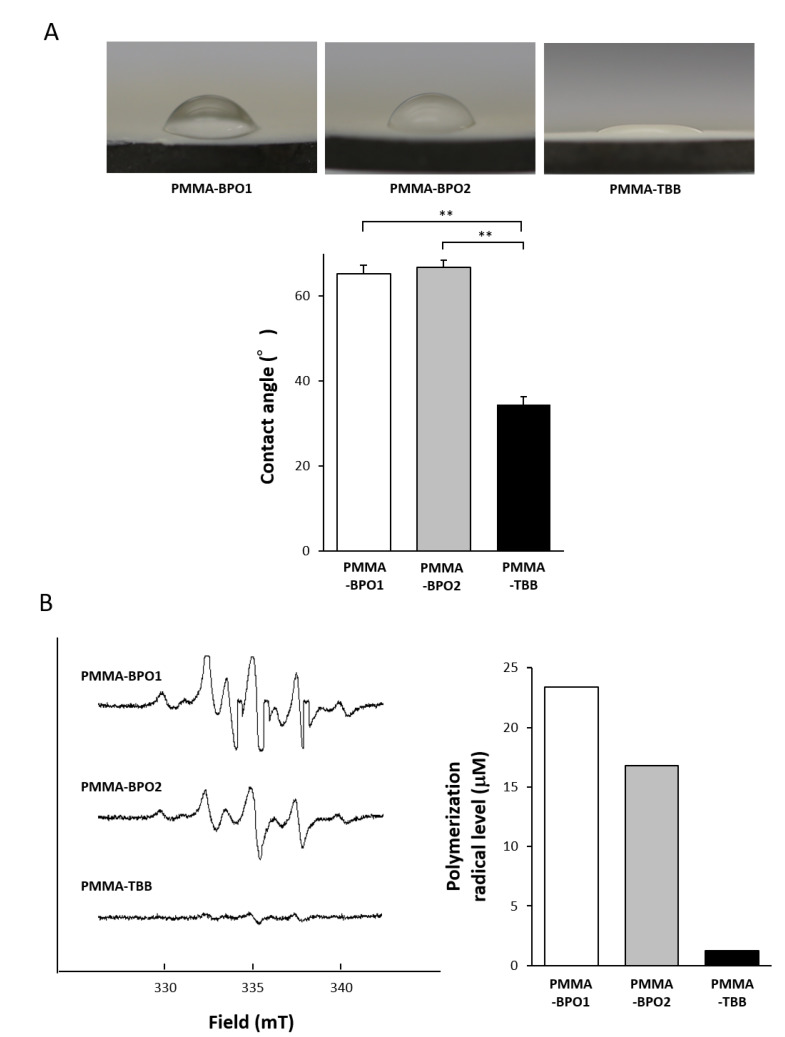
Characteristics of the contact angle and free radical generation on the different bone cement surfaces. (**A**) Hydrophilic/hydrophobic property of the surfaces of the three different materials. Bird’s eye view images of 10 µL ddH_2_O placed on the materials after mixing the materials. The graph on the bottom represents the average contact angle of ddH_2_O for each surface. Each value represents the mean ± standard deviation of triplicate experiments (*n* = 3). ** *p* <0.01, one-way ANOVA followed by a Bonferroni test. (**B**) Free radical generation in polymerizing three different materials evaluated using electron spin resonance spectroscopy (ESR). ESR spectrums recorded 24 h after mixing materials.

The hydrophilic/hydrophobic state was evaluated on the three materials by measuring the contact angle of 10 μL ddH_2_O placed on the material surfaces. The contact angle was greater than 60 °C on the PMMA-BPO cements, indicating that the surfaces were hydrophobic (defined as a contact angle of 45 °C or higher), whereas it was approximately 35 °C on the PMMA-TBB cements, indicating that the surface was hydrophilic (Figure 6A).

Lastly, we examined the amount of polymerization free radical production using electron spin resonance (ESR). The ESR spectrum 24 h after mixing showed a great contrast between the PMMA-TBB and PMMA-BPO cements (Figure 6B). There was a clear detection of free radicals for the PMMA-BPO cements, whereas the PMMA-TBB did not show an identifiable signal. The level of free radical production quantified from the spectra was lower in the order of PMMA-TBB, PMMA-BPO2, and PMMA-BPO1. The radical production within the PMMA-TBB was 1/15–1/20 of that within the PMMA-BPO cements.

### 2.6. Mitigated Toxicity of Bone Cement Materials by Radical Scavenger

**Figure 7:**
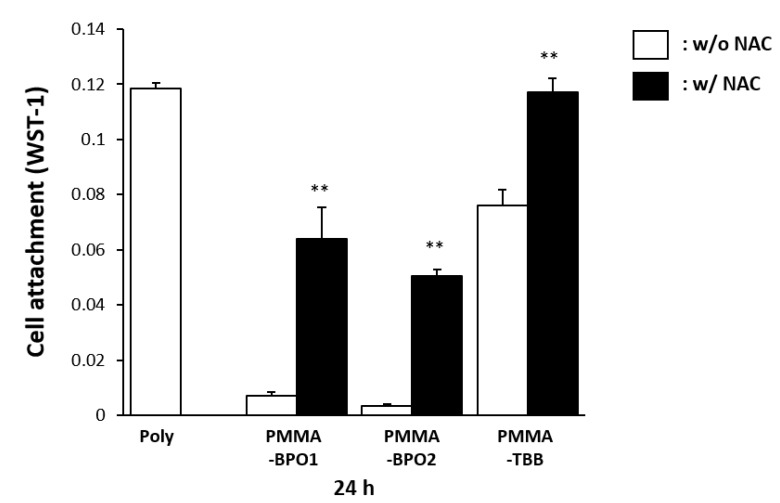
A rescue attempt of three different materials by incorporating the antioxidant amino acid derivative, N-acetyl cysteine (NAC), into the materials examined by the ability of the materials to facilitate cell attachment. The number of osteoblasts attached 24 h after seeding evaluated using WST-1 assay is shown with or without NAC. Each value represents the mean ± standard deviation of triplicate experiments (*n* = 3). ** *p* < 0.01, Welch’s t-test.

We lastly examined how bone cement materials respond to the addition of free radical scavenging molecules. The antioxidant amino acid, N-acetyl cysteine (NAC), was added to each material. Then, the WST-1 assay was performed 24 h after seeding the osteoblasts. The addition of NAC drastically increased the number of cells attached on both PMMA-BPO cements (Figure 7). The number of cells attached to PMMA-TBB, which was high without NAC, did not increase as much as that of the PMMA-BPO cements, although it was of note that the WST-1 value with NAC was as high as the one in the polystyrene dish without a cement. The values for the PMMA-BPO cements with NAC were very similar to the values for the PMMA-TBB without NAC.

## 3. Discussion

This study revealed an improved biocompatibility of the PMMA-TBB material compared to the PMMA-BPO materials selected as representative commercial bone cement products. There was a significant improvement, starting with the number of attached osteoblasts, the initial interaction between the cells and materials, and the growth and ending with their differentiation and mineralization. After 24 h of incubation, the number of cells that survived and were attached on the PMMA-TBB was 10 times greater than that on the PMMA-BPO materials. The greater biocompatibility of the PMMA-TBB material continued to be effective after day 1, resulting in a remarkable increase in the number of propagated cells on day 3. In addition, the result of WST-1 on day 3 was more pronounced than the microscopic observation on the same day. Although there were more cells on the PMMA-TBB than on the PMMA-BPO materials under microscopy, the difference was not as significant as the WST-1 result. The WST-1 assay had to indicate the number of cells by measuring their metabolic activity and was used to evaluate the number of initially attached cells on the surface or the rate of cellular proliferation depending on the culture stage. We assumed that the cells which survived on the PMMA-BPO materials were in the reduced metabolic state, thereby lowering the number of WST-1 results. Indeed, the cells on the PMMA-TBB appeared much larger with more intense expression of cytoskeletal actin, whereas the cells on PMMA-BPO remained smaller and circular, supporting the assumption.

The improved response, behavior, and function of osteoblasts on the PMMA-TBB material were primarily attributed to the reduced toxicity of the material. We found a drastic decrease in the production of polymerization radicals within the PMMA-TBB compared with the PMMA-BPO cements. This decrease was greater than our expectation and as significant as 1/15–1/20 after 24 h. The production of radicals continued to be robust within the PMMA-BPO even after 24 h. The effect of free radicals triggering cellular apoptosis and necrosis and further undermining cellular proliferation and differentiation has been extensively reported. The amount of residual monomer, as another potential source of chemical toxin, was not assessed in the present study and is our future interest. Unreacted monomer and its continuous release as a result of depleted BPO, which has been unavoidable thus far, has been repeatedly reported as a major drawback of current PMMA bone cements [39,50]. If the use of TBB instead of BPO reduces the amount of residual monomer, it would be of additional significance. In addition, we cannot preclude the positive impact of not having N,N-dimethyl-p-toluidine (DmpT) in the PMMA-TBB material. DmpT is a remnant chemical in the polymerized bone cement and is eluted to the surrounding tissue, causing various cellular damages [41,47,51,52]. The PMMA-TBB used in the present study was successfully polymerized without DmpT.

The experiment with NAC further confirmed the adverse effects of free radicals. NAC is known as an effective radical scavenger and mitigated the toxicity of PMMA-BPO cements considerably. Notably, PMMA-BPO cements with NAC turned out to be as biocompatible as the PMMA-TBB. These results re-affirmed that these radicals are the major source of toxicity in PMMA-BPO materials and that a significant control in radical production in the PMMA-TBB material is the reason for its minimum toxicity.

The mechanisms behind radical production are distinctive between BPO and TBB. BPO is highly reactive and degrades readily. In the bone cement products, BPO and DmpT react, cleaving the oxygen single bond (O–O), and form two different radicals (toluidine free radical and benzoyloxy free radical). These two radicals then merge to form a phenyl radical that works as an activated initiator. In contrast, TBB possesses two active boron-carbon bonds, each of which produces radicals by reacting with oxygen, without the aid of DmpT [48,49,53].

We found that other physicochemical properties were significantly different between the PMMA-TBB and PMMA-BPO materials. The exothermic reaction was well controlled in the PMMA-TBB material. The present result was consistent with a previous study reporting that the activation energy (Ea) for BPO (15 to 33 kcal/mol) was significantly higher than that for TBB (3.8 to 5.5 kcal/mol) [54]. The experimental conditions, such as measuring temperature in water and using a smaller material volume than the product size, may have caused a lower temperature reading than the actual ones. However, we believe that the difference of the peak temperature between 29 °C for the PMMA-TBB and 36 °C for the PMMA-BPO is worth the attention. We interpret that the use of TBB as an initiator dissipates thermal energy and enables wide-spread polymerization kinetics, thereby preventing a spike of temperature increase. Traumatic changes of bone tissue occur when they come into contact with bone cement reaching 50 °C for 1 min [55,56]. There is a certain temperature range required to trigger thermal injury and the critical thermo-tolerance temperature may be 43 °C at the cellular level [57]. When thermal injury occurs on osteogenic cells, it induces apoptosis and initiates the bone remodeling cascade [58]. It will be of great importance to determine whether the PMMA-TBB material can avoid in vivo pathologic changes by its polymerization temperature in a future study. In particular, antibiotics containing bone cements have become common recently, and their effectiveness is known to be susceptible to the temperature of the bone cement [2,59].

The PMMA-TBB material may have another impact. Another notable change found on the PMMA-TBB was its hydrophilicity. The effect of hydrophilicity or hydrophobicity on cellular attachment was extensively studied on titanium [60,61,62,63,64]. In general, hydrophilic titanium attracts more osteoblasts than hydrophobic titanium. However, no definitive correlation was found between the degree of hydrophilicity and the number of cells. To the best of our knowledge, the effect of hydrophilicity has rarely been studied on bone cement materials [27]. Making hydrophilic PMMA-based materials is challenging in the first place. Now that the use of TBB can potentially make the PMMA surface hydrophilic, further studies will be necessary to pursue its role.

**Figure 8:**
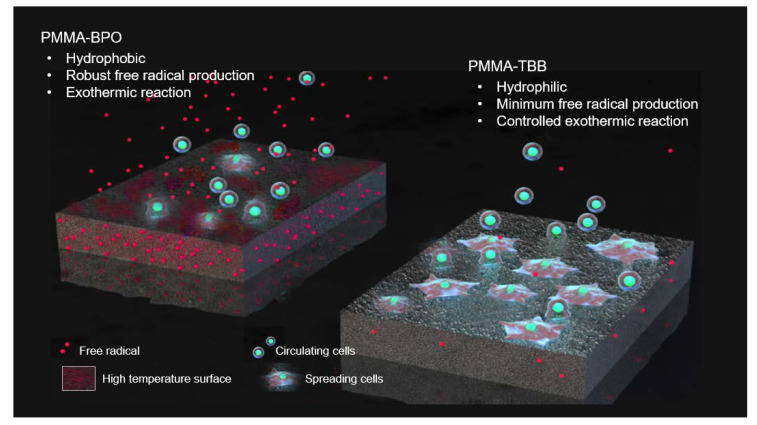
Schematic diagram of the unique physicochemical property of polymethyl methacrylate- tri-n-butyl borane (PMMA-TBB), characterized by a hydrophilic surface, minimum free radical production, and controlled exothermic reaction during polymerization, which enhanced cell attachment and subsequent osteogenic function.

As summarized in Figure 8, a newly tested PMMA-TBB material, compared with the PMMA-BPO material, was characterized by a hydrophilic surface, controlled increase in the surface temperature, and reduced free radical production. Although the detailed link of these likely advantages to its biological potential remains to be confirmed, PMMA-TBB materials may warrant further in vivo studies as well as their mechanical characterization, in pursuit of their application to orthopedic bone cements. The pursuit may extend the acrylic bone cement per se. We believe the fundamental principles and biological advantages of TBB found in the present study can be applied to various PMMA-based materials loaded with other ingredients. Specifically, the effect of the use of TBB on antibiotic-impregnated bone cements [1,65,66,67] and calcium phosphate/hydroxyapatite and PMMA composites [68,69,70,71] should be examined. Further, comparisons between PMMA-TBB materials and currently used calcium phosphate bone cements and ceramic-based materials will be of great interest.

## 4. Materials and Methods

### 4.1. Material Preparation and Characterization

PMMA-BPO1 bone cement was prepared by mixing the powder and liquid in the recommended ratio (powder (wt):liquid (wt) ratio = 40:18.88; Endurance, DePuy Orthopaedics, Warsaw, Indiana) and placed in a well of a 12-well cell culture plate. PMMA-BPO2 bone cement was also prepared by mixing powder and liquid in the recommended ratio (powder (wt):liquid (vol) ratio = 40:20; Surgical Simplex P, Stryker, Kalamazoo, Michigan). For PMMA-TBB resin, TBB initiator was added to the MMA monomer in a ratio of 9% to make a liquid mix. Then, the PMMA powder and liquid were mixed in the ratio (wt/wt) 40:18.8. The MMA, PMMA, and TBB materials were manufactured and provided by Mitsui Chemical Inc. (Tokyo, Japan). Ingredients for the power and liquid for each material are listed in Table 1. Three bone cement specimens were prepared for each assay per cement type.

To determine the role of free radical production on the biocompatibility of the PMMA materials, we prepared the experimental PMMA materials supplemented with N-acetyl-cysteine (NAC). NAC is a cysteine derivative and is known to be an antioxidant amino acid by directly scavenging free radicals [7,17,22,23,72]. NAC was prepared as a 1M stock solution in HEPES buffer whose pH was adjusted to 7.2. For each material, the NAC solution was mixed with MMA liquid before mixing with the PMMA powder to make a final concentration of 5 mM NAC.

Potential temperature increase of the materials was evaluated by measuring the temperature during polymerization. Each cement was mixed and placed in a well of a 12-well plate submerged in 500 μL PBS pre-warmed to 37 °C. The measurement was conducted every minute until a peak-out. The hydrophobic/hydrophilic property of the material surfaces was examined by measuring the contact angle of 10 μL ddH_2_O placed on the materials. The measurement was performed on a material surface spread flat after passing their doughy stage.

The production of free radicals during polymerization was assessed using electron spin resonance spectroscopy (ESR), which has been validated and developed for various in vitro biomedical applications [73,74,75]. Cement specimens were examined using a JES-RE 3X, X-band spectrometer (JEOL, Tokyo, Japan) connected to a WIN-RAD ESR Analyzer (Radical Research, Tokyo, Japan) at the following settings: modulation amplitude, 0.063 mT; sweep width, 5 mT; sweep time, 1 min; time constant, 0.03 sec; microwave power, 8 mW; and magnetic field, 335.5 mT. The component signals in the spectra were identified and quantified as reported previously [73]. The measurement was conducted 1 h after mixing.

### 4.2. Osteoblastic Cell Culture

Bone marrow cells isolated from the spines of 8-week-old male Sprague-Dawley rats were placed in alpha-modified Eagle’s medium supplemented with 15% fetal bovine serum, 50 μg/mL ascorbic acid, 10^−8^ M dexamethasone, 10 mM Na-ß-glycerophosphate, and antibiotic-antimycotic solution containing 10,000 units/mL penicillin G sodium, 10,000 mg/mL streptomycin sulfate and 25 mg/mL amphotericin B. Cells were incubated in a humidified atmosphere of 5% CO_2_ at 37 °C. At 80% confluency, the cells were detached using 0.25% trypsin-1 mM EDTA-4Na and seeded onto either PMMA-BPO or PMMA-TBB material at a density of 6 × 10^4^ cells/cm^2^. The culture medium was renewed every 3 days. All experiments were performed following protocols approved by The Chancellor’s Animal Research Committee at the University of California at Los Angeles (ARC #2005-175-41E, approved on 30 January 2018), the PHS Policy for the Humane Care and Use of Laboratory Animals, and the UCLA Animal Care and Use Training Manual guidelines.

### 4.3. Quantification of Cell Number

The number of cells attached to the bone cement materials was evaluated by measuring the number of cells attached to the surfaces after 1 and 3 days of incubation. These measurements were performed using a water-soluble tetrazolium salts (WST-1)-based colorimetric assay (WST-1; Roche Applied Science, Mannheim, Germany). Each culture well was incubated at 37 °C for 30 min with 100 μL WST-1 reagent. The amount of formazan produced was measured at 420 nm using an enzyme-linked immunosorbent assay (ELISA) reader (Synergy HT, BioTek Instruments, Winooski, VT, USA). Three bone cement specimens were prepared for each cement type at each time point.

### 4.4. Morphology and Spreading Behavior of Osteoblasts

The spreading behavior and cytoskeletal arrangement of osteoblasts seeded onto bone cement materials were examined using fluorescence microscopy. At 3 days after seeding, cells were fixed in 10% formalin and dual stained with fluorescent dyes: 4′,6-Diamidino-2-phenylindole (DAPI, Vector, CA, USA) for nuclei and rhodamine phalloidin for actin filaments (Molecular Probes, Eugene, OR, USA). The area, perimeter, and Ferret’s diameter were quantified using an image analyzer (ImageJ, NIH, Bethesda, MD, USA). Three bone cement specimens were prepared for each cement type. Cytomorphometry was conducted in six cells randomly chosen from these specimens.

### 4.5. Alkaline Phosphatase (ALP) Activity

The ALP activity of osteoblasts was examined on day 7 using a colorimetry-based assay. The culture was rinsed with double-distilled water (ddH_2_O) and treated with 250 µL p-nitrophenylphosphate (LabAssay ALP, Wako Pure Chemicals, Richmond, VA, USA) and further incubated at 37 °C for 15 min. ALP activity was evaluated as the amount of nitrophenol released through the enzymatic reaction and measured at a wavelength of 405 nm using an ELISA plate reader. Three bone cement specimens were prepared for each cement type.

### 4.6. Real-Time Quantitative Polymerase Chain Reaction (qPCR)

Gene expression was analyzed using qPCR on day 7. Total RNA was extracted from cells using TRIzol (Invitrogen, Carlsbad, CA, USA) and a Direct-zol RNA MiniPrep kit (Zymo Research, Irvine, CA, USA). Extracted RNA was reverse transcribed into first-strand cDNA using SuperScript III Reverse Transcriptase (Invitrogen). Quantitative PCR was performed in a 20 μL volume containing 90 ng cDNA, 10 μL TaqMan Universal Master Mix II, and 1 μL TaqMan Gene Expression Assay using a QuantStudio 3 Real-Time PCR System (Thermo Fisher Scientific, Canoga Park, CA, USA), to quantify the expression of type I collagen and osteocalcin mRNA. *Gapdh* expression was used as the endogenous control. Three bone cement specimens were prepared for each cement type.

### 4.7. Mineralizing Capability

The mineralization capability of cells was examined by visualizing mineralized nodule areas via Alizarin red staining. On day 14 of culture, the culture was washed twice with 1× PBS at 37 °C and stained for 15 min using 1% Alizarin red (pH 6.3–6.4). The culture wells were then rinsed twice with ddH_2_O and air-dried. The area of mineralized nodules relative to the culture area was quantified using an image analyzer. Three bone cement specimens were prepared for each cement type.

### 4.8. Statistical Analysis

ANOVA was used to determine differences between the three bone cement types, followed by a Bonferroni post-hoc test. In addition, Welch’s *t*-test was used to compare the two groups; *p* < 0.05 was considered statistically significant.

## 5. Conclusions

The PMMA-TBB material allowed the attachment of a greater number of osteoblasts and more advanced spread, proliferative activity, and differentiation of the cells than the PMMA-BPO bone cements tested in this study. This highly improved biocompatibility of the PMMA-TBB was associated with its distinct physicochemical properties, including a hydrophilic surface, controlled exothermal polymerization, and minimal production of free radicals.

## Figures and Tables

**Table 1:** Bone cement materials compared in the present study.

Bone Cement Type	Ingredients
PMMA-BPO1(Endurance MV, DePuy Orthopaedics)	Powder: Polymethyl methacrylate (PMMA) (67.05%) Methyl methacrylate/styrene copolymer (21.10%) Benzoyl peroxide (BPO) initiator (1.85%) Barium sulphate (10.00%) Liquid: Methyl methacrylate (MMA) (98.00%) N,N-dimethyl-p-toluidine (DmpT)(<2.00%) Hydroquinone (HQ) (75 ppm)
PMMA-BPO2(Surgical Simplex P, Stryker)	Powder: Polymethyl methacrylate (PMMA) (15.00%) Methyl methacrylate-styrene copolymer (73.70%) Benzoyl Peroxide (BPO) initiator (1.30%) Barium sulfate (10.00%) Liquid: Methyl methacrylate (MMA) (97.40%) N,N-dimethyl p-toluidine (DmpT) (2.60%) Hydroquinone (HQ) (75 ppm)
PMMA-TBB(Experimental)	Powder: Polymethyl methacrylate (PMMA) (90.00%) Barium sulfate (10.00%) Liquid: Methyl methacrylate (MMA) (91.00%) tri-n-butyl borane (TBB) initiator (9.00%) Hydroquinone (HQ) (50 ppm)

## Abbreviations

ALP	alkaline phosphatase
BCIS	bone cement implantation syndrome
BPO	benzoyl peroxide
cDNA	complementary DNA
DAPI	4′,6-diamidino-2-phenylindole
ddH_2_O	double-distilled H_2_O
DmpT	N,N-dimethyl-p-toluidine
ELISA	enzyme-linked immunosorbent assay
ESR	electron spine resonance
MMA	methyl methacrylate
NAC	N-acetyl-cysteine
PCR	polymerase chain reaction
PMMA	polymethyl methacrylate
ROS	reactive oxygen species
TBB	tri-n-butyl borane
WST	water-soluble tetrazolium salts
